# A Fluorescent Composite of Carbon-Dot-Embedded Covalent Organic Frameworks for Highly Sensitive and Rapid Detection of Biogenic Amines in Large Yellow Croaker

**DOI:** 10.3390/foods15081449

**Published:** 2026-04-21

**Authors:** Yunying Xia, Han Wu, Xin You, Haofeng Huang, Zhiming Yan, Zhihui Luo, Qinghua Yao, Hui Xu

**Affiliations:** 1 Engineering Research Centre of Fujian-Taiwan Special Marine Food Processing and Nutrition, Ministry of Education, Fuzhou 350002, China; 52309010076@fafu.edu.cn (Y.X.); 52309010037@fafu.edu.cn (H.W.); 3210910056@fafu.edu.cn (X.Y.); 15770612868@163.com (H.H.); fjyzm@fafu.edu.cn (Z.Y.); 2 College of Food Science, Fujian Agriculture and Forestry University, Fuzhou 350002, China; 3 Guangxi Key Laboratory of Agricultural Resources Chemistry and Biotechnology, College of Chemistry and Food Science, Yulin Normal University, Yulin 537000, China; zhluo@ylu.edu.cn; 4 Institute of Quality Standards Testing Technology for Agro-Products, Fujian Academy of Agricultural Sciences, Fuzhou 350003, China; yaoqh24@163.com

**Keywords:** biogenic amines, covalent organic framework, carbon dots, fluorescent probes, large yellow croaker

## Abstract

The excessive accumulation of biogenic amines (BAs) in aquatic products poses serious health risks, necessitating the development of rapid and sensitive detection methods. This study reports the synthesis of a novel fluorescent nanocomposite, carbon-dot-embedded covalent organic frameworks (CDs@COFs). Comprehensive characterization (TEM, XPS, FTIR, UV–Vis, and fluorescence spectroscopy) confirmed the successful fabrication of the nanocomposites, which exhibited excellent thermal and optical stability. A significantly enhanced quantum yield of 36.22% (compared with 12.92% for pure carbon dots) was obtained. As a fluorescent probe, the composite enabled the detection of nine BAs based on a fluorescence quenching mechanism. The proposed method demonstrated good linearity (1~100 ng/mL) and low detection limits of 0.58~0.98 ng/mL. The method was successfully applied to analyze tyramine in large yellow croaker, showing accurate spike recoveries ranging from 91.93% to 101.43% and excellent reproducibility (RSD < 3%). These results highlight the great potential of the developed method as a powerful tool for the rapid screening of BAs in aquatic products.

## 1. Introduction

Biogenic amines (BAs) are a class of nitrogen-containing aliphatic or heterocyclic low-molecular-weight compounds, which are primarily generated through the decarboxylation of free amino acids, catalyzed by decarboxylases secreted by microorganisms [1]. Common BAs include putrescine, cadaverine, tryptamine, tyramine, β-phenylethylamine, histamine, octopamine, spermidine, and spermine [2]. Moderate levels of BAs have physiological regulatory effects on the human body [3], but excessive intake may lead to toxic reactions, which can be life-threatening in severe cases [4]. Food regulatory authorities in various countries primarily set limit standards for histamine content in fish and fish products. For example, the U.S. Food and Drug Administration requires a histamine limit of 50 mg/kg for high-histamine fish [4]; the European Union stipulates that histamine levels in food must not exceed 100 mg/kg; in China, histamine limits are set at 400 mg/kg and 200 mg/kg depending on the fish species [5]. As an important economic fish species in China, large yellow croaker is susceptible to BA formation during storage, which is attributed to microbial activity and enzymatic reactions. This not only affects product quality but may also pose a threat to consumer health [6]. Therefore, the establishment of accurate and reliable BA detection methods is of great practical significance for ensuring the safety and quality control of large yellow croaker products.

Currently, the main detection methods for biogenic amines include high-performance liquid chromatography (HPLC) [7], HPLC–mass spectrometry (HPLC-MS) [8], gas chromatography–mass spectrometry (GC-MS) [9], capillary electrophoresis (CE) [10], electrochemical sensors [11], and enzyme-linked immunosorbent assay (ELISA) [12]. Although these methods can achieve accurate quantification of BAs, they generally have inherent limitations, such as high instrument costs, tedious and time-consuming sample pretreatment procedures, and difficulties in target enrichment. These drawbacks render them unsuitable for large-scale sample screening and on-site detection [13]. In recent years, nanomaterial-based fluorescence sensing technology has attracted considerable attention in the field of analytical detection due to its advantages of simple operation, rapid response, and high sensitivity [14], thereby providing new perspectives for the detection of BAs.

Carbon dots (CDs) have exhibited unique advantages in food safety detection, which are attributed to their excellent fluorescence properties, good biocompatibility, and tunable surface chemistry [15]. CD-based fluorescent probe technology has been successfully applied to BA detection in food matrices [16]. Meng et al. [13] constructed a dual-emission nano fluorescent probe by covalently modifying methyl red on CD surfaces, enabling rapid detection of tyramine, tryptamine, and dimethylamine within 30 s. Yan et al. [17] developed a dual-emission ratiometric fluorescent sensor by mixing blue-fluorescent CDs with yellow-fluorescent CdTe quantum dots for rapid quantification of eight BAs. Yang et al. [18] designed the ratiometric fluorescent intelligent labels, where CDs served as pH indicators and protoporphyrin IX (PpIX) as an internal reference, exhibiting the best selectivity for tyramine.

However, nano-sized CDs (especially those <10 nm) easily undergo fluorescence quenching due to self-aggregation, impairing their fluorescence performance [19]. CDs are typically encapsulated in three-dimensional cross-linked networks or porous materials to prevent aggregation. Covalent organic frameworks (COFs) possess a porous layered structure, low density, high specific surface area, and excellent thermal stability, that make them ideal for target enrichment [20]. Notably, Feng et al. [21] designed a cationic C—C single-bond-linked COF, which achieved improved affinity with alkaline amines through Lewis acid–base interactions, thus serving as an ideal sensing platform for the selective detection of ammonia and amines. Xie et al. [22] proposed azide-functionalized covalent organic frameworks confined in molecularly imprinted polymers, which realized the rapid detection of tryptamine in meat products. Therefore, encapsulating CDs within porous COFs not only avoids CD aggregation but also enhances target enrichment via the intrinsic porous structure of COFs, representing an ideal strategy for improving fluorescence sensing performance. Recently, researchers have attempted to combine CDs with COFs for detection applications. Zhang et al. [23] encapsulated CDs in covalent organic frameworks (TDCOFs) and grafted thermoresponsive poly(N-isopropylacrylamide) (PNIPAM) to construct CDs@TDCOFs@PNIPAM fluorescent material for temperature-responsive “on/off” detection of pyrethroids. Song et al. [24] wrapped N-doped carbon dots (N-CDs) in COFBTT-Th nanosheets to prepare NCD nanosheets with strong dual-emission fluorescence, significantly improving COFBTT-Th’s fluorescence performance. Qin et al. [25] employed imine-based COFs as supporting material and CDs as fluorescent sensing elements to prepare TpPa-1 COF@CD fluorescent composite material via inverse emulsion polymerization, which was successfully applied to the adsorption and detection of Hg^2+^.

Herein, nitrogen-doped carbon dots (N-CDs) were synthesized via a solvent-free method. The N-CDs were embedded into the COF matrix, yielding composite nanomaterial N-CDs@COFs. In this composite system, N-CDs serve as sensing elements that provide strong and stable fluorescence signals, while COFs, leveraging their high specific surface area, ordered porous structure, and specific functional group interactions, enable efficient adsorption and enrichment of BAs. This integration not only resolves the self-aggregation problem of CDs but also enhances the targeting and sensitivity of BA detection. The synergistic effect of CDs and COFs makes the composite probe possess the characteristics of rapid response, high sensitivity, and good stability for BA detection, thereby providing a reliable technical tool for aquatic product food safety monitoring. The proposed method is superior to the single-component sensing materials reported previously and the traditional BA detection methods requiring complex pretreatment procedures.

## 2. Materials and Methods

### 2.1. Materials and Reagents

Fresh large yellow croaker samples were purchased from the local market in Fuzhou City, Fujian Province, China. The following reagents were used: citric acid, potassium chloride, ethanol, methanol, acetone, hydrochloric acid, sodium hydroxide, and sodium chloride (Sinopharm Chemical Reagent Co., Ltd., Shanghai, China); urea (Aladdin Reagent, Shanghai, China); p-toluenesulfonic acid (97%), 2-nitro-1,4-phenylenediamine (95%), 1,3,5-triformylphloroglucinol (97%), acetonitrile (analytical grade), N,N-dimethylformamide, metronidazole (99%), nitrofurantoin (98%), cadaverine (98%), β-phenylethylamine (98%), tryptamine (98%), tyramine (98%), spermine (98%), spermidine (99%), histamine (96%), putrescine dihydrochloride (99%), and octopamine hydrochloride (98%) (Shanghai Macklin Biochemical Technology Co., Ltd., Shanghai, China); acetic acid (HPLC grade) (Shanghai Eonchem Technology Co., Ltd., Shanghai, China); 0.1 M PBS buffer (pH = 8) (Shanghai Yeasen Biotechnology Co., Ltd., Shanghai, China); sulfadiazine (1000 μg/mL) and sulfathiazole (1000 μg/mL) (Mannhag (Shanghai) Biotechnology Co., Ltd., Shanghai, China); five heterocyclic amine single standards (100 μg/mL) (Alta Scientific Co., Ltd., Tianjin, China); proline (98%), glycine (98%), histidine (98%), glutamic acid (98%), arginine (98%), lysine (98%), tyrosine (98%), and tryptophan (98%) (Beijing Solarbio Science & Technology Co., Ltd., Beijing, China); purified water (Hangzhou Wahaha Group Co., Ltd., Hangzhou, China).

### 2.2. Synthesis of Fluorescent Nanocomposite

The synthesis of N-CDs was carried out following a previously reported method [26] with minor modifications. Briefly, 0.01 mol of citric acid (CA), 0.04 mol of urea, and 0.025 mol of potassium chloride were thoroughly ground, followed by a reaction in an autoclave at 180 °C for 4 h. The resulting product was dispersed in a 10% ethanol/water solution, subjected to centrifugal washing, and freeze-dried to obtain a dark-brown N-CD powder. The fluorescent nanocomposite was synthesized by modifying the preparation protocol of COFs (TpPa-NO_2_) [27]. Specifically, 0.2764 mmol of *p*-toluenesulfonic acid, 0.045 mmol of 2-nitro-1,4-phenylenediamine (Pa-NO_2_), and 0.0375 mmol of 1,3,5-triformylphloroglucinol (TP) were placed in a 15 mL centrifuge tube. Then, 10 mL of acetonitrile and 50 mg of N-CDs were added, and the mixture was thoroughly homogenized by ultrasonication until complete dissolution. Subsequently, 0.7 mL of 12 M acetic acid was added to the mixture, which was vortexed for 10 s and then allowed to stand at room temperature for 72 h. Upon completion of the reaction, the reddish-brown precipitate was collected by centrifugation at 10,000 rpm for 6 min and washed three times with N,N-dimethylformamide, acetone, and deionized water sequentially to remove excess impurities and unreacted carbon dots. Finally, the product was vacuum-dried at 60 °C for 24 h to obtain N-CD-embedded COFs (N-CDs@TpPa-NO_2_).

### 2.3. Characterization of Synthetic Materials

The morphology and structure of the synthesized materials were characterized by transmission electron microscopy (TEM) (JEM-2100, JEOL Ltd., Tokyo, Japan), scanning electron microscopy (SEM) (ZEISS Sigma 300, Carl Zeiss AG, Oberkochen, Germany), thermogravimetric analysis (TGA) (TG/DTA8122, Rigaku Corporation, Tokyo, Japan), Brunauer–Emmett–Teller (BET) surface area and porosity analysis (APSP 2460, Micromeritics Instrument Corporation, Norcross, GA, USA), X-ray diffraction (XRD) (Ultima IV, Rigaku Corporation), Fourier-transform infrared spectroscopy (FTIR) (iN10, Thermo Scientific, Waltham, MA, USA), and X-ray photoelectron spectroscopy (XPS) (K-Alpha, Thermo Scientific, USA).

### 2.4. Optical Performance Analysis of N-CDs@TpPa-NO_2_

The absorption peaks of the materials were characterized using a UV–visible spectrophotometer (NANODROP 2000C, Thermo Scientific, USA); the excitation and emission peaks were measured using a fluorescence spectrophotometer (RF-6000, Shimadzu Corporation, Kyoto, Japan); and the quantum yield was determined using a steady-state/transient fluorescence spectrometer (FLS 1000, Edinburgh Instruments Ltd., Edinburgh, UK). Additionally, time-correlated single-photon counting (TCSPC) was employed to measure the decay time of two systems: N-CDs@TpPa-NO_2_ without bioamine (N-CDs@TpPa-NO_2_) and N-CDs@TpPa-NO_2_ with bioamine (N-CDs@TpPa-NO_2_ + bioamine). The decay curves were fitted using a double-exponential function. The average lifetime *τ*_ave_ (ns) of the system was calculated, with the relevant formula as follows. Here, *R*(*t*) is the sum of the single exponential decay intensities, *B*_1_ and *B*_2_ are the pre-exponential factors, and *τ*_1_ and *τ*_2_ are the decay times (ns).

(1) $$R\left(t\right)=B_{1}\exp\left(-\frac{t}{\tau_{1}}\right)+B_{2}\exp\left(-\frac{t}{\tau_{2}}\right)$$

(2) $$\tau_{ave}=\frac{B_{1}\tau_{1}^{2}+B_{2}\tau_{2}^{2}}{B_{1}\tau_{1}+B_{2}\tau_{2}}$$

### 2.5. Biogenic Amine Detection with N-CDs@TpPa-NO_2_

For the fluorescence detection experiment, 40 μL of BA standard solutions with different concentrations were added to 10 μL of N-CDs@TpPa-NO_2_ solution, and the mixture was thoroughly homogenized. Then, 350 μL of 0.1 M phosphate-buffered saline (PBS) buffer solution (pH = 8) was added, followed by thorough mixing. The resulting mixture was incubated at 30 °C for 9 min, and the fluorescence emission spectrum was recorded under excitation at 350 nm. All detection measurements were performed in triplicate for each concentration. The limit of detection (LOD) was calculated using the following formula:

(3) $$LOD=\frac{3SD}{\left|k\right|}$$

where *LOD* is the limit of detection (ng/mL), *SD* is the standard deviation of blank measurements, and *k* is the slope of the calibration curve.

### 2.6. Specificity Investigation of N-CDs@TpPa-NO_2_ for BA Detection

To evaluate the specificity of N-CDs@TpPa-NO_2_ for BA detection, 17 potential interfering substances were selected, including metronidazole, nitrofurantoin, sulfadiazine, sulfathiazole, 2-amino-3,4,8-trimethylimidazo[4,5-f]quinoline, 2-amino-3,8-dimethylimidazo[4,5-f]quinoline, 2-amino-3,4-dimethylimidazo[4,5-f]quinoline, 2-amino-3,7,8-trimethylimidazo[4,5-f]quinoline, 2-amino-1-methyl-6-phenylimidazo[4,5-b]pyridine, proline, glycine, histidine, glutamic acid, arginine, lysine, tyrosine, and tryptophan. Two comparative systems were established: (1) 10 μL of N-CDs@TpPa-NO_2_ solution mixed with 40 μL of different interfering substance solutions, and (2) the same as (1) but with the additional addition of 40 μL of BA standard solution. Both systems were diluted to a final volume of 400 μL with 0.1 M PBS buffer (pH 8.0), ensuring that final concentration of each interfering substance was 20 ng/mL. Subsequently, the fluorescence emission spectra of both systems were measured under excitation at 350 nm. All detection measurements were performed in triplicate for each sample.

### 2.7. Validation of N-CDs@TpPa-NO_2_ for BA Detection in Real Croaker Samples

Commercially available fresh large yellow croakers from Fuzhou City, Fujian Province, China, were selected as actual test samples. The edible parts of the croakers were cleaned and homogenized to obtain the original sample for BA analysis. To verify the reliability of the proposed method, spike recovery experiments were conducted by spiking BA standard solutions at concentrations of 5, 10, and 15 ng/mL into the large yellow croaker matrix, respectively. Methanol was then added to the mixture, which was vortex-mixed for 30 min, followed by centrifugation at 2000 r/min for 5 min. The supernatant was collected, and the extraction process was repeated twice. The combined supernatants were used as the test sample. The BA content in the large yellow croaker samples was determined according to the method described in Section 2.5. All detection measurements were performed in triplicate for each concentration and sample. The spike recovery rate was calculated according to the following formula:

(4) $$R(\%)=\frac{C_{measured}-C_{original}}{C_{spiked}}\times 100\%$$

where *R* is the spike recovery rate (%), *C*_measured_ is the measured concentration of spiked sample (ng/mL), *C*_original_ is the concentration of original sample (ng/mL), and *C*_spiked_ is the concentration of spiked sample (ng/mL).

## 3. Results and Discussion

### 3.1. Characterization and Analysis of Synthesized Materials

**Figure 1:**
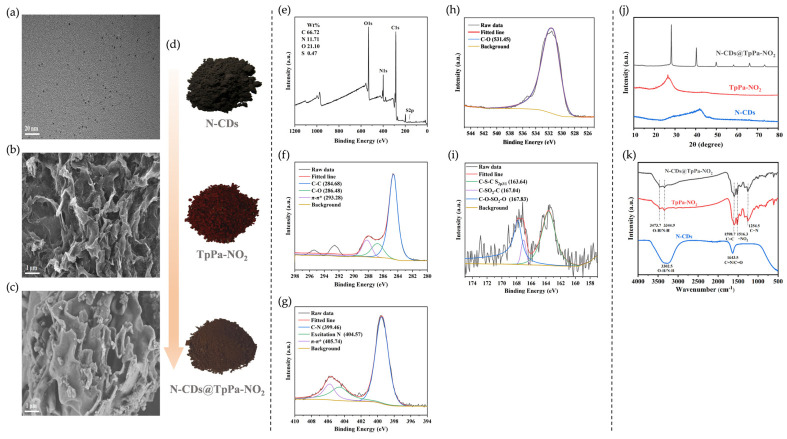
Characterization of N-CDs, TpPa-NO_2_ and N-CDs@TpPa-NO_2_. (**a**) TEM image of N-CDs; (**b**) SEM image of TpPa-NO_2_; (**c**) SEM image of N-CDs@TpPa-NO_2_; (**d**) photographs of the three materials; (**e**–**i**) XPS spectra of N-CDs@TpPa-NO_2_: (**e**) survey spectrum, (**f**) C1s, (**g**) N1s, (**h**) O1s, and (**i**) S2p high-resolution spectra; (**j**) XRD patterns; (**k**) FT-IR spectra. Note. Abbreviations: N-CDs = nitrogen-doped carbon dots, TpPa-NO_2_ = covalent organic frameworks (COFs), N-CDs@TpPa-NO_2_= N-CD-embedded COFs.

The N-CDs were characterized by TEM. As shown in Figure 1a, the N-CDs exhibited as uniformly dispersed quasi-spherical nanoparticles with an average particle size of 2.64 ± 0.33 nm. The morphological structures of TpPa-NO_2_ and N-CDs@TpPa-NO_2_ were observed by SEM. As illustrated in Figure 1b, TpPa-NO_2_ displayed a flower-like porous layered structure, which was attributed to the π-π stacking interactions within its interlayer structure [28]. N-CDs@TpPa-NO_2_ (Figure 1c) retained a similar morphology feature. However, irregular spherical structures were observed on its surface and within the interlayer pores, indicating the successful encapsulation of N-CDs into TpPa-NO_2_ through strong physical adsorption. Figure 1d shows photographic images of the three synthesized materials. The dark-red TpPa-NO_2_ and the dark-brown N-CDs combined to form brown N-CDs@TpPa-NO_2_, demonstrating distinct color differences. Figure 1e shows the XPS survey spectrum of N-CDs@TpPa-NO_2_, where four characteristic peaks corresponding to C1s (284.82 eV), N1s (398.44 eV), O1s (531.87 eV), and S2p (164.97 eV) can be observed, with elemental contents of 66.72%, 11.71%, 21.10%, and 0.47%, respectively. In the high-resolution C1s spectrum of N-CDs@TpPa-NO_2_ (Figure 1f), the functional groups C–C, C–O, and π–π* are identified at binding energies of 284.68 eV, 286.48 eV, and 293.28 eV, respectively. The N1s spectrum (Figure 1g) is deconvoluted into three peaks at 399.46 eV, 404.57 eV, and 405.74 eV, which are assigned to C–N, excited N, and π–π* [29], respectively. Fitting of the O1s spectrum (Figure 1h) reveals a dominant peak at 531.45 eV, corresponding to the C=O bond. The S2p spectrum (Figure 1i) exhibits characteristic peaks at 163.64 eV, 167.04 eV, and 167.83 eV, attributed to S2p^3/1^C–S–C, C–SO_2_–C, and C–O–SO_2_–O [30], respectively. These results confirm the successful synthesis of N-CDs@TpPa-NO_2_. The materials were characterized by XRD as shown in Figure 1j. N-CDs exhibited a broad diffraction peak at 2θ = 42°, which could be assigned to the (100) characteristic diffraction peak of graphitic carbon structure [28]. TpPa-NO_2_ displayed a characteristic diffraction peak at 2θ = 26.6°, primarily attributed to the π-π stacking between two-dimensional layers of COFs, corresponding to the (001) plane, indicating its medium crystallinity [31]. For N-CDs@TpPa-NO_2_, characteristic diffraction peaks of N-CDs and TpPa-NO_2_ appeared at 40.3° and 28.2°, respectively, along with new diffraction peaks at 50°, 58.4°, 66°, and 73.4°. Both the emergence of these new peaks and slight shifts in characteristic peak angles were considered related to synthesis conditions. XRD results demonstrated that after introducing N-CDs into the TpPa-NO_2_ framework, the composite maintained its original crystal structure while achieving enhanced crystallinity and improved stability, which agreed well with thermogravimetric characterization results. As shown in Figure 1k, the FT-IR spectrum of N-CDs displayed a broad peak at 3302.5 cm^−1^ attributable to the stretching vibrations of O-H and N-H bonds [32], while the absorption peak at 1643.5 cm^−1^ was associated with stretching vibrations of C=O and C=N double-bond groups [23,33]. TpPa-NO_2_ and N-CDs@TpPa-NO_2_ exhibited similar FT-IR spectra without generation of new functional groups during the reaction. The absorption peaks at 3473.7 cm^−1^ and 3344.9 cm^−1^ were assigned to O-H and N-H stretching vibrations, respectively, whereas the strong absorption peaks at 1598.7 cm^−1^ and 1254.5 cm^−1^ corresponded to C=C and C-N stretching bands, respectively, with the peak at 1516.3 cm^−1^ characteristic of the -NO_2_ functional group [27]. These results indicated that the incorporation of N-CDs did not disrupt the original chemical bonds, and the composite N-CDs@TpPa-NO_2_ maintained its initial covalent structure.

The TG and DTG curves of N-CDs are shown in Figure S1a. When heated from room temperature to 800 °C, the TG curve exhibited a gradual decreasing trend, indicating good thermal stability of N-CDs. The DTG curve revealed two weight loss stages: a minor weight loss below 100 °C, which was attributed to evaporation of residual water and organic solvents adsorbed on the surface of N-CDs, and an approximately 10% weight loss around 560 °C, resulting from the decomposition of the crystalline structure of N-CDs. For TpPa-NO_2_ (Figure S1b), the first weight loss occurred below 110 °C, which was caused by the evaporation of residual organic solvent. This was followed by a significant weight loss of approximately 20% between 300~350 °C, which was ascribed to the collapse of the covalent organic framework structure. For N-CDs@TpPa-NO_2_ (Figure S1c), a 3% weight loss was observed below 100 °C, which was due to solvent evaporation. A cumulative weight loss of 30% occurred in three stages between 200~500 °C, resulting from the partial collapse of the COF framework, with the decomposition of the crystalline structure occurring above 500 °C. The weight loss behavior of N-CDs@TpPa-NO_2_ was similar to that of N-CDs, with the total weight showing a gradual decreasing trend. Only 40% decomposition was observed at 800 °C, suggesting that the internal crystalline structure of TpPa-NO_2_ was changed after encapsulation of N-CDs, thereby enhancing its thermal stability. The nitrogen adsorption–desorption isotherms of TpPa-NO_2_ and N-CDs@TpPa-NO_2_ are shown in Figure S2a and S2b, respectively. Both samples exhibited type IV isotherms with capillary condensation and hysteresis loops, indicating their typical microporous characteristics [34]. The pore size distribution of TpPa-NO_2_ (Figure S2c) revealed pores predominantly distributed between 0~20 nm, with the highest proportion of micropores around 2 nm and an average pore size of 15.4074 nm. In contrast, N-CDs@TpPa-NO_2_ showed an increased average pore size of 23.5880 nm (Figure S2d), likely due to the filling of TpPa-NO_2_ micropores by 2~3 nm N-CDs. BET analysis demonstrated that TpPa-NO_2_ had a specific surface area of 204.1592 m^2^/g and total pore volume of 0.786391 cm^3^/g (Figure S2e), while these values decreased to 30.3658 m^2^/g and 0.179067 cm^3^/g for N-CDs@TpPa-NO_2_ (Figure S2f). This reduction was primarily caused by N-CDs occupying the original nanopores, further confirming the successful incorporation of N-CDs into the framework channels of TpPa-NO_2_ to form the N-CDs@TpPa-NO_2_ composite.

### 3.2. Optical Properties of N-CDs@TpPa-NO_2_

**Figure 2:**
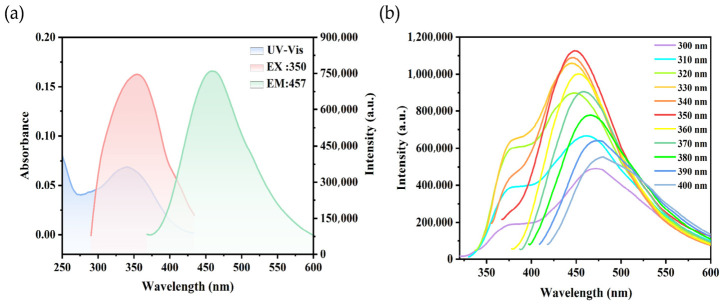
Ultraviolet absorption spectrum (blue), excitation spectrum (red) and emission spectrum (green) of N-CDs@TpPa-NO_2_ (**a**) and fluorescence emission spectra of N-CDs@TpPa-NO_2_ (**b**). Note. Abbreviations: N-CDs@TpPa-NO_2_ = N-CD-embedded COFs.

The optical properties of N-CDs@TpPa-NO_2_ were investigated using ultraviolet–visible (UV–Vis) absorption and fluorescence spectroscopy. As shown in Figure 2a, the UV–Vis absorption spectrum of N-CDs@TpPa-NO_2_ exhibited two distinct absorption peaks at 250 nm and 350 nm. The peak at 250 nm can be attributed to the typical π-π* transition of aromatic sp^2^ domains in the material, while the peak at 350 nm is generally associated with the n-π* transition of nitrogen-doped unsaturated groups, which generates strong emission due to surface-state trapping of excited-state energy [35]. The overlap between the UV absorption peak and excitation peak at 350 nm indicates that the emission peak at 457 nm under 350 nm excitation originates from the n-π* transition. Further analysis of the fluorescence emission spectra of N-CDs@TpPa-NO_2_ under excitation wavelengths ranging from 300 to 400 nm (Figure 2b) revealed that the emission intensity initially increased and then decreased with increasing excitation wavelength, reaching its maximum at 350 nm. Additionally, the emission peak exhibited a gradual red shift with increasing excitation wavelength, demonstrating an excitation-dependent behavior, which may be attributed to differences in energy state distribution caused by varying carbon cluster sizes and surface functional groups [36]. The side peaks observed under 300~340 nm excitation can be ascribed to molecular fluorescence from amorphous aggregates in the material [37]. Since N-CDs@TpPa-NO_2_ exhibited the strongest and most well-defined emission peak under 350 nm excitation, this wavelength was selected as the optimal excitation wavelength, with 457 nm as the optimal emission wavelength. Furthermore, steady-state/transient fluorescence spectroscopy measurements revealed that the quantum yield (QY) of N-CDs@TpPa-NO_2_ under this excitation wavelength reached 36.22%, which was significantly higher than that of N-CDs alone (QY = 12.92%). This result confirms that the encapsulation strategy effectively enhances the fluorescence performance of the material.

### 3.3. Detection Mechanism of N-CDs@TpPa-NO_2_ for BAs

**Figure 3:**
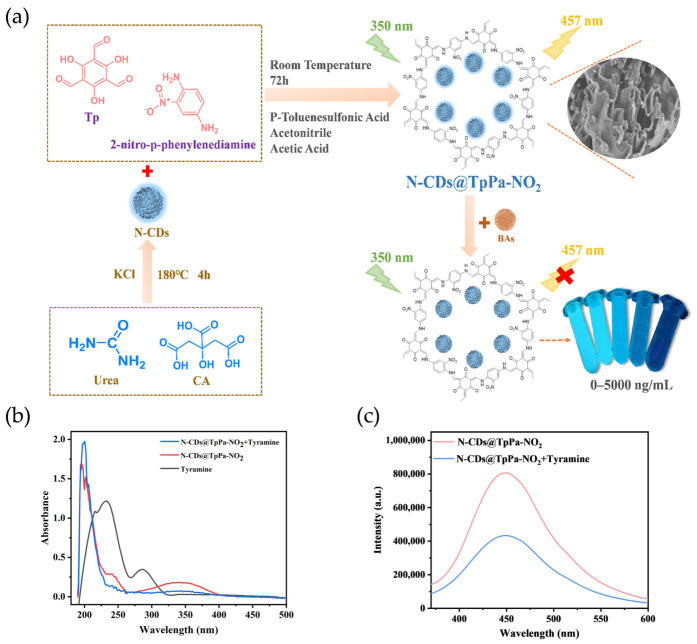
Schematic illustration of the synthesis of N-CDs@TpPa-NO_2_ and its application for biogenic amine detection (**a**); UV–Vis absorption spectrum of N-CDs@TpPa-NO_2_ (**b**); fluorescence emission spectra of N-CDs@TpPa-NO_2_ before and after addition of tyramine (**c**). Note. Abbreviations: Tp = 1,3,5-triformylphloroglucinol, CA = citric acid, N-CDs@TpPa-NO_2_ = N-CD-embedded COFs, BAs = biogenic amines.

The procedure for detecting BAs using N-CDs@TpPa-NO_2_ is illustrated in Figure 3a. The efficient capture of BAs by N-CDs@TpPa-NO_2_ relies on the synergistic effect of physical and chemical adsorption, laying a foundation for fluorescence detection. Nitrogen adsorption–desorption experiments confirm the nanocomposite exhibits a typical type IV isotherm with a hysteresis loop, featuring a microporous structure (average pore size: 23.5880 nm) that provides sufficient diffusion channels and physical adsorption sites for BA molecules, enhancing the effective contact area. FT-IR and XPS characterizations verify its surface is rich in functional groups (-NO_2_, -NH, C=N, C=O, -OH) and framework structures (C–SO_2_–C, C–O–SO_2_–O), which serve as core sites for chemical adsorption. The strong electron-withdrawing -NO_2_ induces electron-deficient centers on adjacent carbons, forming dipole–dipole interactions and charge transfer complexes with -NH_2_ in biogenic amines. Electronegative atoms (N, O) in -NH, C=N, C=O, and O atoms in SO_2_ moieties form hydrogen bonds with active hydrogen in BAs. The layered crystalline structure (XRD characterization) enables π-π stacking between aromatic rings in the framework and BAs, while hydrophobic interactions between the main framework of nanomaterials and aliphatic chains of BAs further enhance adsorption stability and specificity.

The detection of BAs by N-CDs@TpPa-NO_2_ is primarily based on a dynamic quenching mechanism, which is confirmed by relevant characterizations using tyramine as a model analyte. UV–Vis absorption spectroscopy (Figure 3b) shows that tyramine exhibits characteristic strong absorption peaks at 232 nm and 288 nm, corresponding to the π-π* transitions of its intramolecular aromatic rings. After binding with N-CDs@TpPa-NO_2_, no new characteristic peaks appear in the absorption spectrum, and it maintains consistency with the absorption band profile of the pure composite material, indicating that no new ground-state complex is formed between them, thus ruling out the possibility of static quenching. Meanwhile, fluorescence spectroscopy tests reveal that tyramine has a significant quenching effect on the characteristic fluorescence emission peak of N-CDs@TpPa-NO_2_ at 457 nm (Figure 3c), and the quenching degree increases regularly with the increase in biogenic amine concentration. This change can be attributed to energy transfer between N-CDs@TpPa-NO_2_ and the target tyramine [38]. Quantitative analysis by steady-state/transient fluorescence spectroscopy further verifies that under excitation at 350 nm and emission at 457 nm, the average fluorescence lifetime (*τ*_ave_) of pure N-CDs@TpPa-NO_2_ is 6.49 ns, which decreases to 5.84 ns after the addition of tyramine with a significantly accelerated fluorescence decay rate (Table S1). This is consistent with the characteristic of shortened lifetime caused by collisions between excited-state fluorescent molecules and quenchers in dynamic quenching [39]. After N-CDs@TpPa-NO_2_ is excited by 350 nm light, electrons transition to the excited state; when biogenic amine molecules are present in the system, they act as fluorescence quenchers to collide with the excited-state composite material. Through energy transfer, the excited-state electrons return to the ground state via non-radiative transition, resulting in a decrease in fluorescence emission intensity. The higher the concentration of biogenic amines, the greater the collision probability and the more significant the quenching degree. Finally, the quantitative detection of biogenic amines is achieved by monitoring the change in fluorescence intensity at 457 nm.

### 3.4. Optical Stability of N-CDs@TpPa-NO_2_

The effect of pH on the optical stability of N-CDs@TpPa-NO_2_ was investigated (Figure S3a). The fluorescence intensity decreased significantly under strongly acidic or alkaline conditions, accompanied by slight red shifts of emission peaks, while relatively strong fluorescence signals were observed in weakly acidic or alkaline environments, with maximum intensity achieved at pH 8. This pH-dependent behavior was attributed to protonation/deprotonation of surface functional groups. Under weakly alkaline conditions, the deprotonation of surface -OH groups enhanced fluorescence through improved monodispersion induced by electrostatic repulsion. In contrast, the protonation of -NH_2_ groups in acidic environments promoted molecular aggregation via hydrogen bonding, leading to fluorescence quenching. These results were consistent with the hydroxyl and amino groups identified by FT-IR and XPS analyses, confirming that the fluorescence properties were regulated by protonation states of surface groups [40]. The influence of ionic strength was examined using NaCl concentration gradients (0~3.0 mol/L) to simulate physiological conditions (Figure S3b). The fluorescence intensity at 457 nm decreased gradually with increasing NaCl concentration, showing only approximately 10% reduction at 3.0 mol/L, indicating excellent photostability under high ionic strength. The photobleaching stability of N-CDs@TpPa-NO_2_ was investigated by varying the UV irradiation time (Figure S3c). After continuous irradiation with a 365 nm UV lamp for 120 min, the fluorescence intensity of the material decreased by approximately 15% within 20~80 min and then leveled off. Even after exposure to high-intensity UV light, N-CDs@TpPa-NO_2_ still exhibits a relatively high fluorescence intensity, indicating that it possesses a certain degree of resistance to photobleaching.

### 3.5. Detection of BAs by N-CDs@TpPa-NO_2_

**Figure 4:**
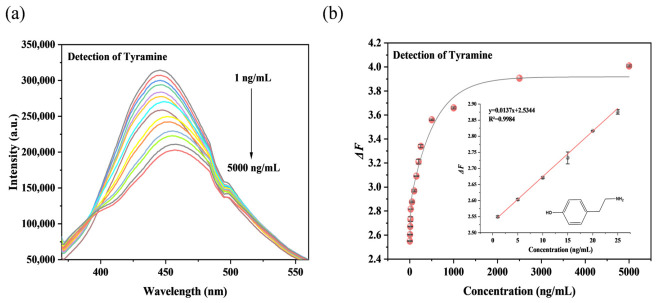
The fluorescence response (**a**) and linear correlation (**b**) of N-CDs@TpPa-NO_2_ under different tyramine concentrations. Note. Abbreviations: N-CDs@TpPa-NO_2_ = N-CD-embedded COFs.

The fluorescent nanomaterial N-CDs@TpPa-NO_2_, composed of nitrogen-doped carbon dots (N-CDs) and the covalent organic framework TpPa-NO_2_, exhibits superior optical properties compared to carbon dots. Therefore, N-CDs@TpPa-NO_2_ can be used as a fluorescent probe to establish a rapid detection method for BAs based on the “signal-off” mode. Taking tyramine as a model analyte, different concentrations of tyramine standard solutions were added to the N-CDs@TpPa-NO_2_ system, and the changes in fluorescence emission spectra under 350 nm excitation were recorded to analyze the linear relationship between tyramine concentration and fluorescence intensity. As shown in Figure 4a, the fluorescence intensity of N-CDs@TpPa-NO_2_ gradually decreased with increasing tyramine concentration, demonstrating effective fluorescence quenching. Figure 4b presents the linear relationship between fluorescence intensity and tyramine concentration, which showed good linearity in the range of 1~25 ng/mL, with a method detection limit of 0.65 ng/mL (equivalent to 4.74 nmol/L).

To further investigate the sensing performance of the N-CDs@TpPa-NO_2_ fluorescent probe for BAs, eight additional common food-borne BAs were tested alongside tyramine, including tryptamine, putrescine, cadaverine, spermine, spermidine, octopamine, β-phenylethylamine, and histamine. The experimental results demonstrated that the probe exhibited excellent response characteristics toward all these BAs. As shown in Figure S4, the fluorescence intensity of N-CDs@TpPa-NO_2_ decreased with increasing concentrations of different BAs, indicating distinct fluorescence quenching effects. Figure S5 revealed significant linear relationships between the fluorescence quenching efficiency (ΔF) and the concentrations of all eight BAs, with detailed linear regression equations, correlation coefficients (R^2^), detection ranges, and detection limits summarized in Table 1. For all nine BAs, the N-CDs@TpPa-NO_2_ fluorescent probe exhibited excellent linearity in the concentration ranges of 1~25 ng/mL and 1~100 ng/mL, with correlation coefficients (R^2^) ranging from 0.9903 to 0.9984 and detection limits between 0.58~0.98 ng/mL (equivalent to 3.62~10.89 nmol/L).

### 3.6. Comparison of N-CDs@TpPa-NO_2_ with Other Methods

Compared with other BA detection methods (Table 2), this method demonstrated lower detection limits, higher sensitivity, and faster response time, indicating that the N-CDs@TpPa-NO_2_ fluorescent probe is suitable for detecting BAs in food products.

### 3.7. Specificity of N-CDs@TpPa-NO_2_ for BA Detection

**Figure 5:**
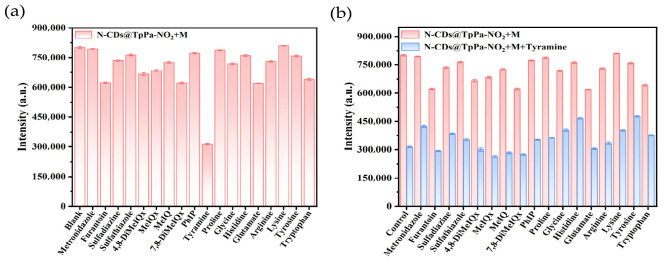
Specificity of N-CDs@TpPa-NO_2_ for biogenic amine detection. (**a**): Addition of 20 ng/mL interfering substance solution to N-CDs@TpPa-NO_2_ solution. (**b**): Addition of both 20 ng/mL interfering substance solution and tyramine solution to N-CDs@TpPa-NO_2_. Note. Abbreviations: N-CDs@TpPa-NO_2_ = N-CD-embedded COFs, M = potential interfering substances.

Using tyramine as a representative analyte, the specificity of N-CDs@TpPa-NO_2_ for BA detection was evaluated against 17 potential interfering substances, which were categorized into three groups: (i) four drug residues (metronidazole, furantoin, sulfadiazine, and sulfathiazole); (ii) five heterocyclic amines (2-amino-3,4,8-trimethylimidazo[4,5-f]quinoline [4,8-DiMeIQx], 2-amino-3,8-dimethylimidazo[4,5-f]quinoline [MeIQx], 2-amino-3,4-dimethylimidazo[4,5-f]quinoline [MeIQ], 2-amino-3,7,8-trimethylimidazo[4,5-f]quinoline [7,8-DiMeIQx], and 2-amino-1-methyl-6-phenylimidazo[4,5-b]pyridine [PhIP]); and (iii) eight amino acids (proline, glycine, histidine, glutamate, arginine, lysine, tyrosine, and tryptophan). As demonstrated in Figure 5a, N-CDs@TpPa-NO_2_ exhibited superior fluorescence quenching toward tyramine compared with other interferents. Figure 5b presents the fluorescence results obtained by the additional introduction of 40 μL tyramine standard solution into each test system under identical conditions. Distinct fluorescence quenching responses were observed across all interference systems following tyramine addition, confirming the excellent specificity and selectivity of N-CDs@TpPa-NO_2_ for BA detection.

### 3.8. Application of N-CDs@TpPa-NO_2_ in Real Croaker Samples

**Figure 6:**
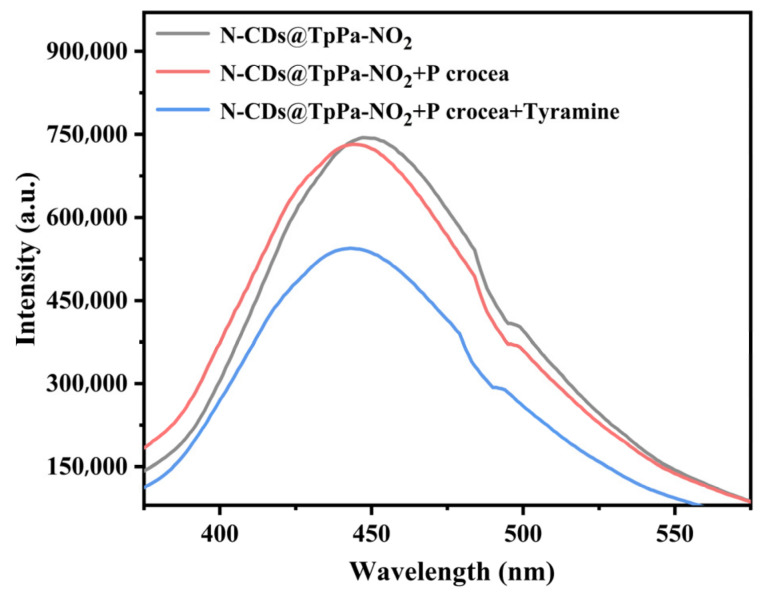
Fluorescence spectra of N-CDs@TpPa-NO_2_ for biogenic amines detection in croaker. Note. Abbreviations: N-CDs@TpPa-NO_2_ = N-CD-embedded COFs.

Fresh croaker samples purchased from markets in Fuzhou were used to validate the practical detection performance of the N-CDs@TpPa-NO_2_ fluorescent probe. With tyramine as the target analyte, Figure 6 displays fluorescence quenching spectra of N-CDs@TpPa-NO_2_ in both fish sample matrix and matrix spiked with tyramine. The results demonstrated that the fluorescence quenching effect of tyramine was minimally affected by the complex fish matrix, indicating excellent anti-interference capability of this method. Subsequently, tyramine levels in three batches of croaker samples (No. 1~3) were determined along with spike recovery tests (Table 3). While no tyramine was detected in Sample 2, the tyramine contents in Samples 1 and 3 were 5.47 ± 0.01 ng/mL and 3.76 ± 0.05 ng/mL, respectively. The spike recovery rates ranged from 91.93% to 101.43%, with relative standard deviations (RSDs) between 0.79% and 2.88%. These results confirm that the N-CDs@TpPa-NO_2_ fluorescent probe is suitable for rapid detection of BAs in croaker, offering high accuracy and precision.

## 4. Conclusions

This study successfully prepared the nanocomposite N-CDs@TpPa-NO_2_ with excellent fluorescent properties, and the established biogenic amines detection method exhibits good linear relationships for nine biogenic amines. Compared with conventional chromatographic techniques and other fluorescent probe methods, this approach features simpler operation, faster response, and higher sensitivity, providing a reliable technical means for the rapid detection of total biogenic amines in croaker and showing significant application potential in food safety monitoring. Despite these merits, mechanistic investigations in this study still have limitations, and subsequent research will employ density functional theory (DFT) calculations and molecular dynamics (MD) simulations to deepen the analysis of microscopic mechanisms including charge transfer and binding configurations. Future work will further extend the application to biogenic amine detection in other food matrices, establish food spoilage kinetic models, optimize the visualization performance of the sensing interface, and develop portable detection devices, promoting the upgrading of food freshness monitoring towards real-time and intelligent directions.

## Figures and Tables

**Table 1:** Linear equation, correlation coefficient, linear range and detection limit of BAs by N-CDs@TpPa-NO_2_ detection. Note. Abbreviations: BAs = biogenic amines, N-CDs@TpPa-NO_2_ = N-CD-embedded COFs.

Analyte	Linear Equation	Correlation Coefficient (R^2^)	Linear Range (ng/mL)	LOD (ng/mL)	LOD (nmol/L)
Tryptamine	y = 0.015x + 1.3343	0.9963	1~25	0.58	3.62
1,4-butanediamine	y = 0.0062x + 1.3304	0.9980	1~25	0.96	10.89
1,5-pentanediamine	y = 0.0084x + 1.5154	0.9979	1~25	0.81	7.93
Spermine	y = 0.0129x + 1.1839	0.9921	1~25	0.78	3.85
Spermidine	y = 0.0144x + 1.1374	0.9903	1~25	0.82	5.65
Tyramine	y = 0.0137x + 2.5344	0.9984	1~25	0.65	4.74
Octopamine	y = 0.004x + 1.4657	0.9943	1~25	0.98	6.40
Phenethylamine	y = 0.0016x + 1.3901	0.9952	1~100	0.93	7.67
Histamine	y = 0.0025x + 1.4119	0.9979	1~100	0.86	7.74

**Table 2:** Comparison with other methods for the detection of BAs. Note. Abbreviations: BAs = biogenic amines.

Method	Materials	Target Analytes	LOD	References
Electrochemical	WS_2_/CS/SPE	Histamine	0.0844 μmol/L	[41]
Electrochemical	Ag-Ag_2_O/MWCNTs/GCE	Histamine	0.18 μmol/L	[42]
Fluorescence	CdTe QDs and N,S CDs	Spermine, Spermidine, 1,4-butanediamine, 1,5-pentanediamine, Histamine, Tyramine, Tryptamine, Phenethylamine	1.259~5.428 μmol/L	[17]
Fluorescence	CMA-Cl	1,5-pentanediamine, 1,4-butanediamine	209 nmol/L	[43]
Fluorescence	PTCN	1,5-pentanediamine	46 nmol/L	[44]
Fluorescence	N-CDs@TpPa-NO_2_	Tryptamine, Tyramine, 1,4-butanediamine, 1,5-pentanediamine, Spermine, Spermidine, Octopamine, Phenethylamine, Histamine	0.58~0.98 ng/mL (equivalent to 3.62~10.89 nmol/L).	This work

**Table 3:** Determination of tyramine in real croaker samples.

Sample	Concentration of Original Sample (ng/mL)	Concentration of Spiked Sample (ng/mL)	Measured Concentration of Spiked Sample (ng/mL)	Recovery (%)	RSD (%)
1	5.47 ± 0.01	5	10.18 ± 0.10	91.93~95.43	0.99
		10	15.41 ± 0.16	96.54~101.33	1.03
		15	19.97 ± 0.20	93.27~97.04	1.01
2	ND	5	4.86 ± 0.08	95.31~98.07	1.62
		10	9.58 ± 0.28	93.34~98.76	2.88
		15	14.63 ± 0.34	95.12~99.70	2.36
3	3.76 ± 0.05	5	8.73 ± 0.08	98.00~101.43	0.98
		10	13.64 ± 0.11	97.03~100.00	0.79
		15	18.48 ± 0.18	96.73~99.81	0.97

## Data Availability

The original contributions presented in this study are included in the article/Supplementary Materials. Further inquiries can be directed to the corresponding author.

## Supplementary Materials

The following supporting information can be downloaded at https://www.mdpi.com/article/10.3390/foods15081449/s1, Figure S1: TG and DTG curve of N-CDs (a), TpPa-NO_2_ (b) and N-CDs@TpPa-NO_2_ (c); Figure S2: N2 adsorption/desorption isotherms (a,b), BJH adsorption dV/dlog(D) pore volume (c,d) and BET surface area plot (e,f) of TpPa-NO_2_ and N-CDs@TpPa-NO_2_; Figure S3: N-CDs@TpPa-NO_2_ emission spectra at different pH values (a), fluorescence intensity at different NaCl concentrations (b), and fluorescence intensity at different UV lamp irradiation time (c); Figure S4: The fluorescence response of N-CDs@TpPa-NO_2_ under different concentrations of Tryptamine (a), 1,4-butanediamine (b), 1,5-pentanediamine (c), Spermine (d), Spermidine (e), Octopamine (f), Phenethylamine (g), Histamine (h); Figure S5: The linear correlation of N-CDs@TpPa-NO_2_ under different concentrations of Tryptamine (a), 1,4-butanediamine (b), 1,5-pentanediamine (c), Spermine (d), Spermidine (e), Octopamine (f), Phenethylamine (g), Histamine (h); Table S1: Bi-exponential fitting parameters of fluorescence decay curves of N-CDs@TpPa-NO_2_ and N-CDs@TpPa-NO_2_+Tyramine. Note. Abbreviations: N-CDs@TpPa-NO_2_= N-CDs embedded COFs.
