# Supplementary material for: A Fluorescent Composite of Carbon-Dot-Embedded Covalent Organic Frameworks for Highly Sensitive and Rapid Detection of Biogenic Amines in Large Yellow Croaker
Source: Foods. 2026 Apr 21;15(8):1449. doi: 10.3390/foods15081449 (PMC13116696; doi:10.3390/foods15081449)
Supplement: Supplementary file 1 [file foods-15-01449-s001.zip › foods-4208532-supplementary.pdf]

foods

Supplementary Materials

A Fluorescent Composite of Carbon Dots-Embedded Covalent Organic Frameworks for Highly Sensitive and Rapid Detection of Biogenic Amines in Large Yellow Croaker

Yunying Xia [1,2], Han Wu [1,2], Xin You [1,2], Haofeng Huang [1,2], Zhiming Yan [1,2], Zhihui Luo [3], Qinghua Yao [4] and Hui Xu [1,2,*]

1 Engineering Research Centre of Fujian-Taiwan Special Marine Food Processing and Nutrition, Ministry of Education, Fuzhou 350002, Fujian, China; 52309010076@fafu.edu.cn (Y.X.); 52309010037@fafu.edu.cn (H.W.); 3210910056@fafu.edu.cn (X.Y.); 15770612868@163.com (H.H.); fjyzm@fafu.edu.cn (Z.Y.)
2 College of Food Science, Fujian Agriculture and Forestry University, Fuzhou 350002, Fujian, China
3 Guangxi Key Laboratory of Agricultural Resources Chemistry and Biotechnology, College of Chemistry and Food Science, Yulin Normal University, Yulin 537000, Guangxi, China; zhluo@ylu.edu.cn (Z.L.)
4 Institute of Quality Standards Testing Technology for Agro-products, Fujian Academy of Agricultural Sciences, Fuzhou 350003, Fujian, China; yaoqh24@163.com (Q.Y.)
* Correspondence: xhuifst@fafu.edu.cn (H.X.)

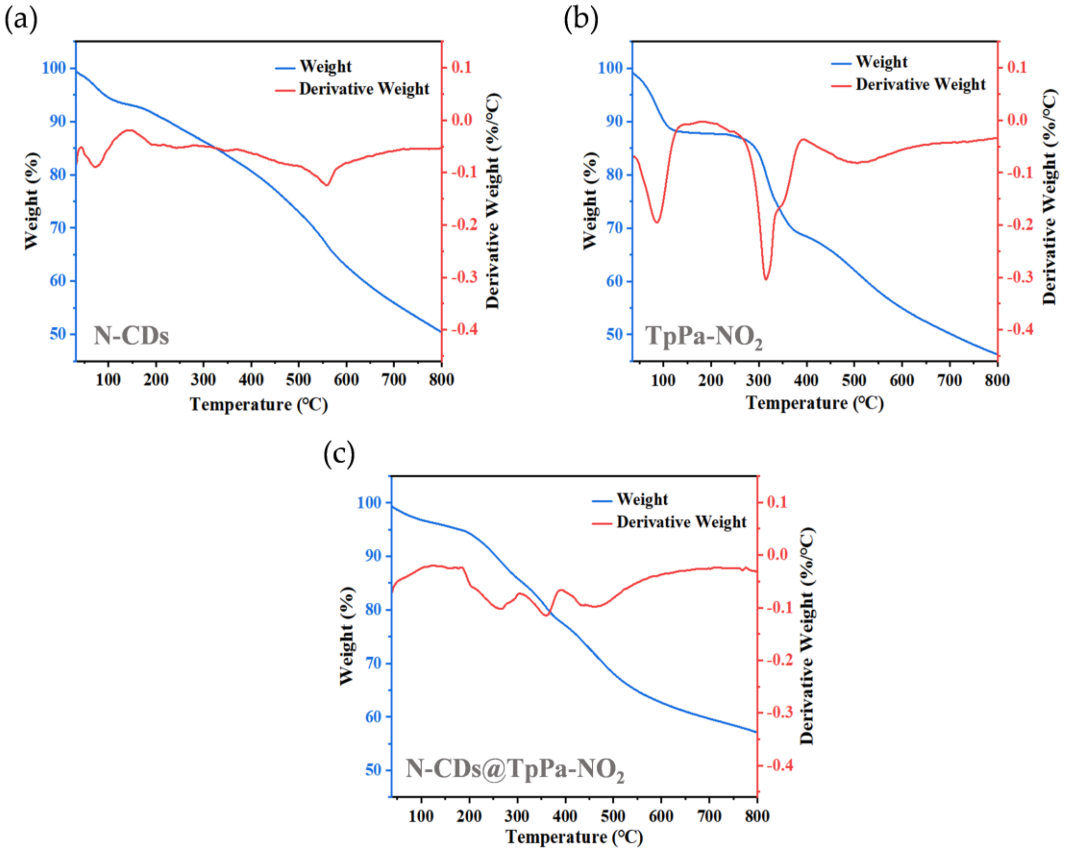

Figure S1. TG and DTG curve of N-CDs (**a**), TpPa-NO_2 (**b**) and N-CDs@TpPa-NO_2 (**c**).

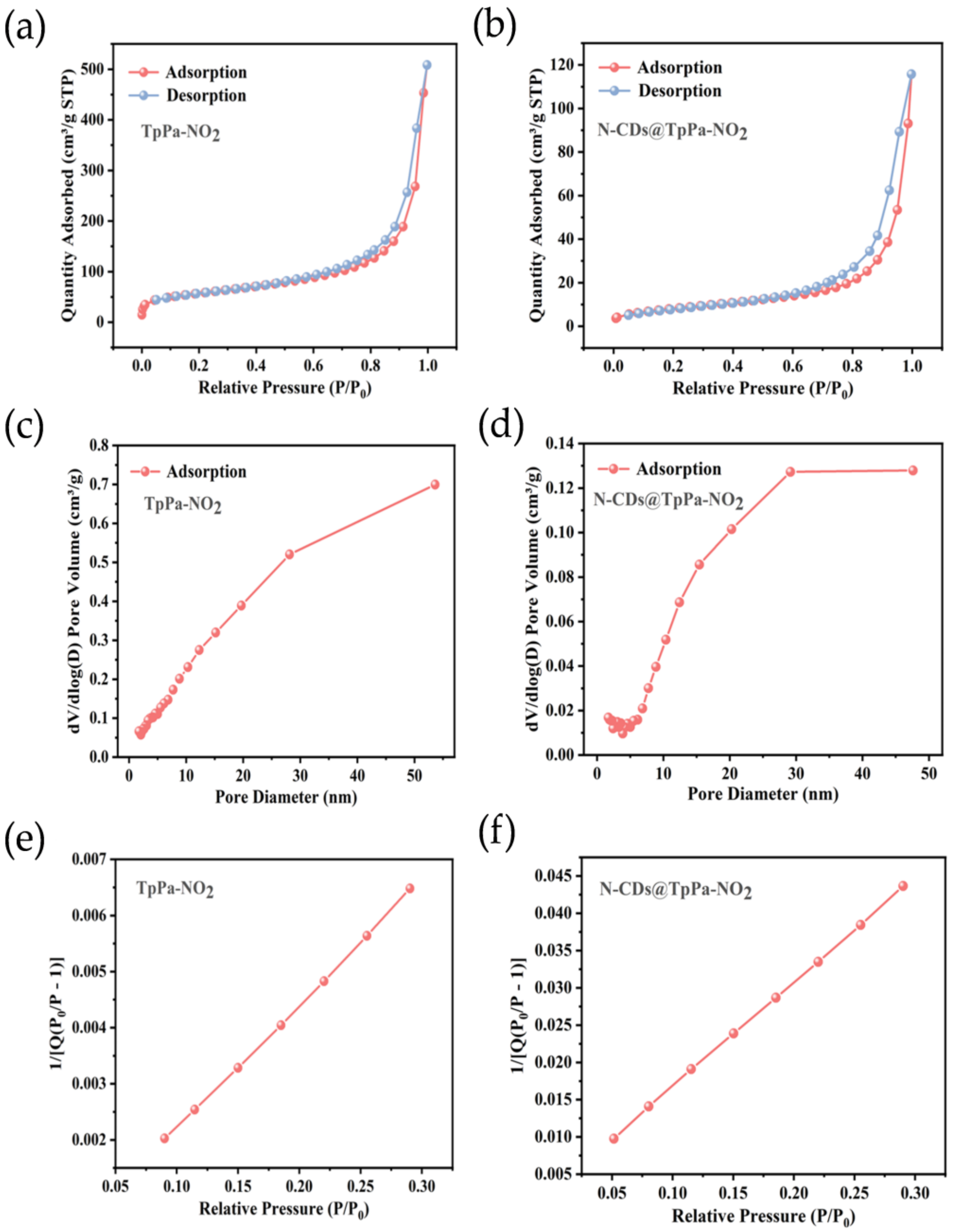

Figure S2. N_2 adsorption/desorption isotherms (**a, b**), BJH adsorption dV/dlog(D) pore volume (**c, d**) and BET surface area plot (**e, f**) of TpPa-NO_2 and N-CDs@TpPa-NO_2.

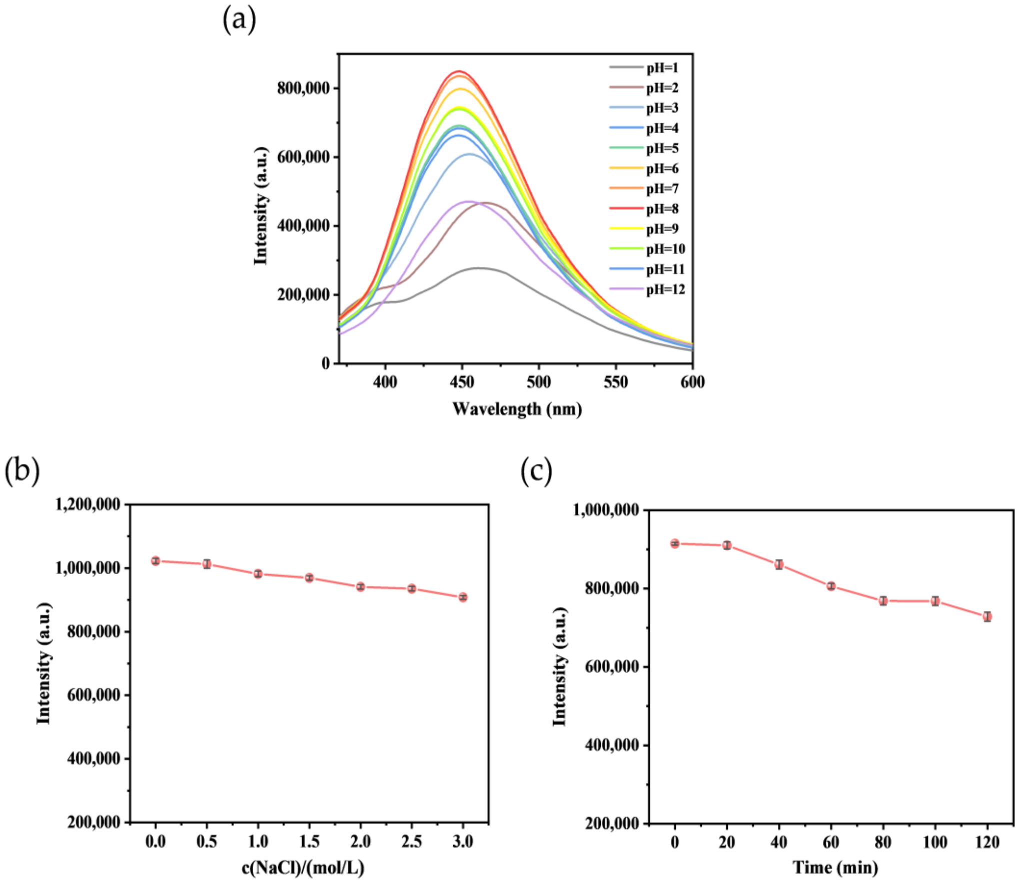

Figure S3. N-CDs@TpPa-NO_2 emission spectra at different pH values (**a**), fluorescence intensity at different NaCl concentrations (**b**), and fluorescence intensity at different UV lamp irradiation time (**c**).

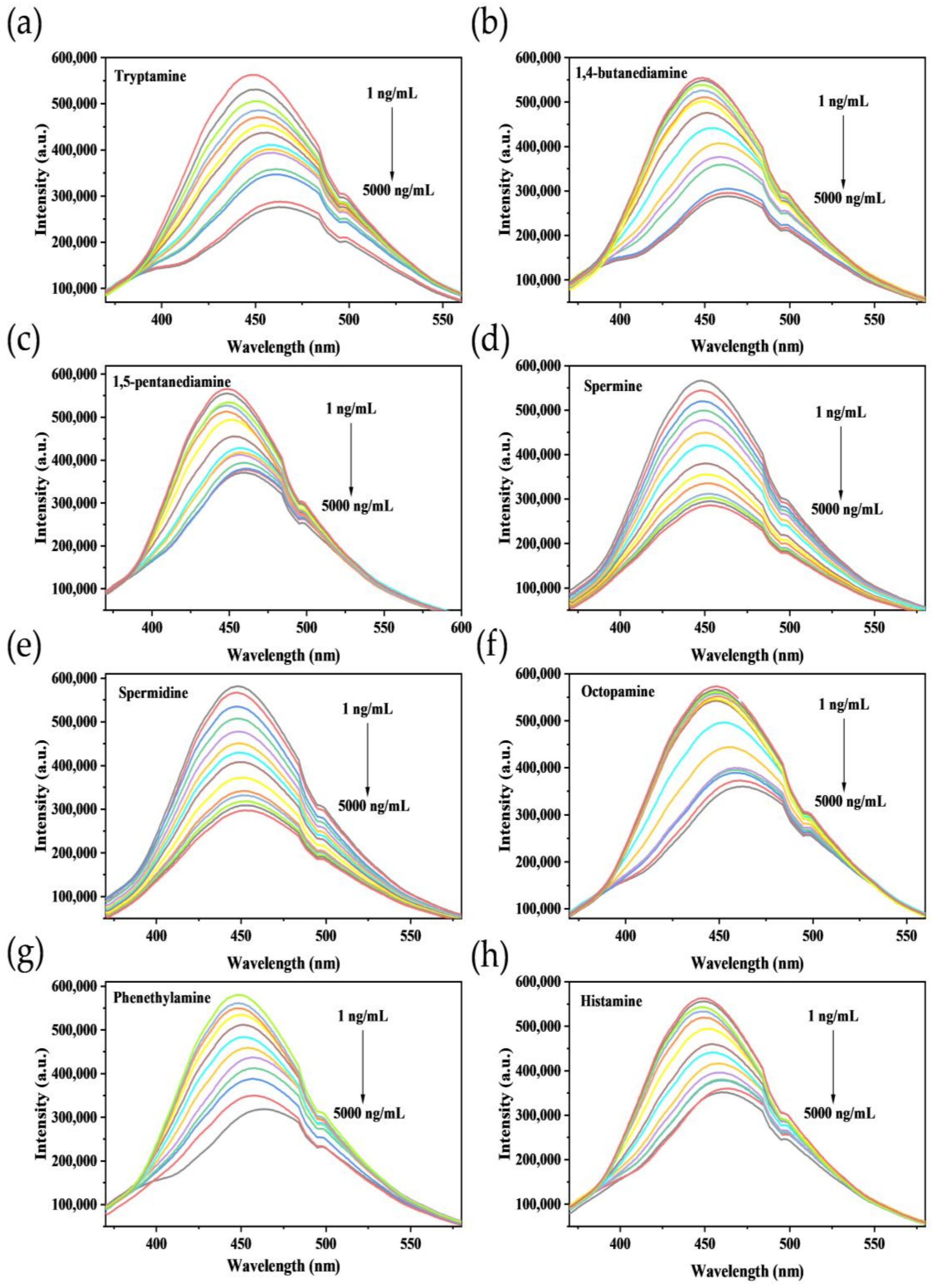

Figure S4. The fluorescence response of N-CDs@TpPa-NO_2 under different concentrations of Tryptamine (**a**), 1,4-butanediamine (**b**), 1,5-pentanediamine (**c**), Spermine (**d**), Spermidine (**e**), Octopamine (**f**), Phenethylamine (**g**), Histamine (**h**).

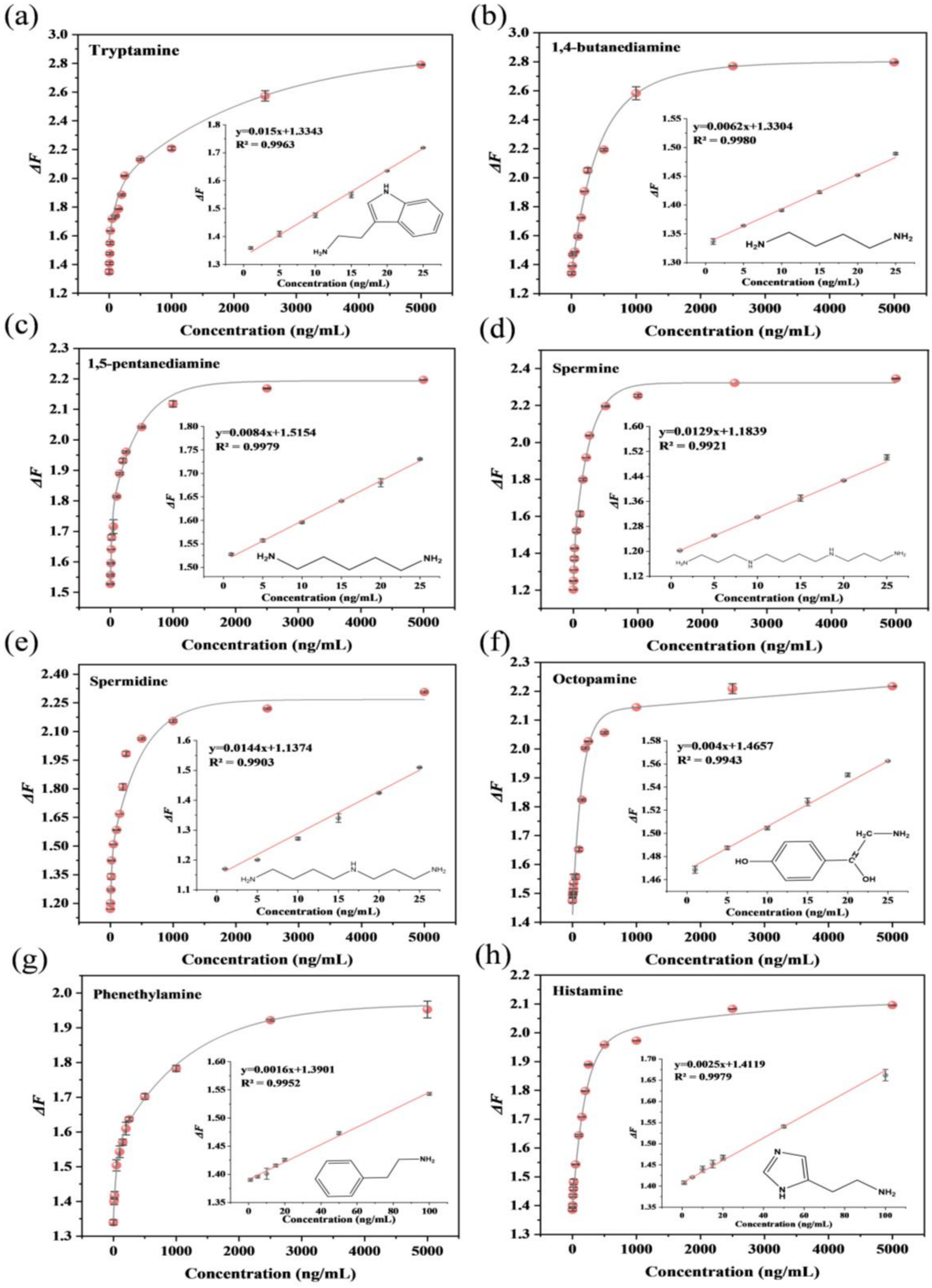

Figure S5. The linear correlation of N-CDs@TpPa-NO_2 under different concentrations of Tryptamine (**a**), 1,4-butanediamine (**b**), 1,5-pentanediamine (**c**), Spermine (**d**), Spermidine (**e**), Octopamine (**f**), Phenethylamine (**g**), Histamine (**h**).

Table S1. Bi-exponential fitting parameters of fluorescence decay curves of N-CDs@TpPa-NO_2 and N-CDs@TpPa-NO_2+Tyramine. Note. Abbreviations: N-CDs@TpPa-NO_2= N-CDs embedded COFs.

**Sample**	B_1	τ_1/ns	B_2	τ_2/ns	τ_{ave} /ns
N-CDs@TpPa-NO_2	10.69	2.66	2.93	10.15	6.49
N-CDs@TpPa-NO_2+Tyramine	9.50	2.80	1.97	9.96	5.84
